# Extensive natural killer cell receptor phenotyping on NK and T cells discloses differences in RA and PsA, potentially mirroring diverse immunoregulatory functions

**DOI:** 10.1186/1479-5876-9-S2-P42

**Published:** 2011-11-23

**Authors:** Sandra TA van Bijnen, Marta Cossu, Frank Preijers, Jan Spanholtz, Harry Dolstra, Timothy RDJ Radstake

**Affiliations:** 1 Dept. of Hematology, Radboud University Nijmegen Medical Centre, Nijmegen, The Netherlands; 2 Dept. of Rheumatology, Radboud University Nijmegen Medical Centre, Nijmegen, The Netherlands; 3 Referral Center for Systemic Autoimmune Diseases, Fondazione IRCCS Ca' Granda Ospedale Maggiore Policlinico, Milan, Italy; 4 Dept. of Laboratory Medicine, Laboratory of Hematology, Radboud University Nijmegen Medical Centre, Nijmegen, The Netherlands

## Introduction

Rheumatoid arthritis (RA) and psoriatic arthritis (PsA) are immune-mediated diseases which partly share clinical features, but differentiate in the mechanisms which lead to aberrant immune responses. Natural killer (NK) cells tune the innate immune response depending on the integration of signals coming from a complex network of activating and inhibitory surface receptors.

## Aim

We aim to elucidate the potential role of NK and T cells in controlling inflammation and autoimmunity in RA and PsA and we focused on extensively characterizing the NK cell receptor (NKR) (co)-expression patterns on their surface.

## Patients and methods

The frequency of T and NK cells expressing Killer like immunoglobulin Receptors (KIR), NKG2 and Natural Cytotoxicity receptors was assessed by 10-color flow cytometry in peripheral blood of 23 RA patients, 12 PsA patients and 18 healthy controls (HC).

## Results

The frequency of NK cells expressing the inhibitory receptor NKG2A is increased in RA patients compared to HC - particularly in patients with rheumatoid factor positivity -, as well as NKG2A^+^ CD158e1e2^-^ NKG2C^-^ and NKG2A^+^ CD158e1e2^+^ NKG2C^+/-^ NK cells. T cells expressing the Fcγ receptor CD16 are higher in RA than in HC. We found higher frequencies of T cells expressing the KIRs CD158ah in RA and PsA and CD158e1e2 in RA compared to HC. CD4^+^ T cells expressing the KIRs CD158ah, CD158b1b2j and CD158e1e2 were low, but significantly elevated compared to HC.

## Conclusions

The differences in NKR expression on NK and T cells in RA and PsA could mirror the diverse pathogenic mechanisms implicated in the two diseases and provide common ground to unravel functional consequences.

